# Accuracy of Mansoura Early Feeding Skills Assessment score (MEFSA) in identification of oral feeding readiness in preterm infants

**DOI:** 10.1186/s12887-026-06586-z

**Published:** 2026-03-18

**Authors:** Omnia El-Kassas, Maii Saad, Ayman Amer, Hesham Abdel-Hady, Tamer Abou-Elsaad

**Affiliations:** 1 Phoniatrics Unit, ORL Department, Faculty of Medicine, Mansoura University, Mansoura, 35516 Egypt; 2 Neonatology Unit, Pediatric Department, Faculty of Medicine, Mansoura University, Mansoura, Egypt; 3 Nile Valley University, Fayoum, Egypt

**Keywords:** Prematurity, Readiness, Oral feeding skills, Scale, Full oral feeding

## Abstract

**Background:**

Oral feeding readiness is a complex concept. More evidence is needed to approach the initiation of oral feedings in preterm infants. Recognition and support of oral feeding readiness may decrease hospital stays and reduce healthcare costs.

**Purpose:**

To assess the accuracy of the pre-feeding section of the Mansoura Early Feeding Skills Assessment (MEFSA) score on the initiation of oral feeding in preterm infants and identify a cutoff point for oral feeding readiness concerning the oral feeding skill (OFS) level. Furthermore, to confirm the concordance between the MEFSA and the OFS level.

**Methods:**

Transversal and analytical study of 41 preterm infants. Infants were assessed before the first oral feeding by the pre-feeding section. Furthermore, the first oral feeding act is evaluated by the during-feeding section of MEFSA and OFS level. The cutoff point was estimated based on the OFS level and success using a receiver operating characteristic (ROC) curve. Pearson correlations were obtained between the pre-feeding and during-feeding sections, as well as the association between the during-feeding section and OFS score.

**Results:**

MEFSA’s overall diagnostic accuracy was 82.5%. Cutoff points for oral feeding readiness were determined to be greater than 77 and greater than 77.5, concerning OFS level and oral feeding success, respectively. A moderate positive correlation coefficient existed between the pre-feeding and during-feeding sections of MEFSA.

**Conclusion:**

The pre-feeding section of MEFSA is a straightforward, quick instrument that can be used in clinical practice. It has a moderate to high accuracy in pinpointing the ideal time for readiness for oral feeding. However, more research is needed. We suggest combining the pre-feeding and during-feeding sections in clinical practice.

**Supplementary Information:**

The online version contains supplementary material available at 10.1186/s12887-026-06586-z.

## Introduction

The medical community caring for preterm infants and medically complex infants struggles with the difficulties of oral feeding [1]. Transitioning from tube feeding to oral feeding is challenging. Clinically, determining the optimal timing for initiating oral feeding is complex and varies across centers [2]. Delayed oral feeding prolongs the discharge of these infants, as independent oral feeding is one of the significant criteria for discharge [1]. Such an occurrence increases medical costs and delays mother-infant reunion [3, 4].

A formal readiness screening instrument has been used to improve the accuracy of determining the appropriate time to initiate oral feedings [5] such as the Neonatal Oral Motor Assessment Scale (NOMAS) [6, 7], The “Oral Feeding Readiness” section of The Early Feeding Skills Assessment (EFS) [8], Premature Oral Feeding Readiness Assessment Scale (POFRAS) [9], Preterm Infant Nipple Feeding Readiness Scale (PINFRS) [5, 10], The Infant Driven Scale (Modified Italian scale) [11] and A Rapid Salivary Proteomic Platform for Oral Feeding Readiness in the Preterm Newborn [12].

The NOMAS is a visual observation tool that assesses both NNS and NS [6, 7, 13]. It is the most widely used in infants under six months to identify and quantify oral motor abilities [14–16]. However, NOMAS is not predictive of oral feeding performance [5]. Additionally, the process of sucking was not objectively evaluated; therefore, incorporating pulse oximetry would have provided a more integrated interpretation [17]. The EFS can be used from the time of SOF and through OFS maturation [8]. It measures physiologic stability, as indexed by sufficient oxygen saturation, but it did not undergo formal content validity testing [18]. POFRAS evaluates SOF readiness using NNS [9]. It is an easy, quick instrument, but evaluating oral feeding readiness based on behavioral data may not guarantee success [19]. PINFRS indirectly measures feeding readiness, but reliability and validation studies of this instrument are still needed [5]. The Infant Driven Scale (Modified Italian scale) appears to be a useful additional instrument for assessing the oral feeding readiness of preterm infants and the early identification of infants at risk for delayed feeding independence [11]. A rapid salivary Proteomic Platform for Oral Feeding Readiness in the Preterm newborn is a panel of five genes required for oral feeding success that were identified in neonatal saliva. It aimed to translate these five transcriptomic biomarkers into a rapid proteomic platform, enabling an objective, real-time assessment of OFS [12]. The OFS levels assessment tool is an objective indicator of infants’ feeding ability, taking into account their skills and endurance [20]. Although this approach has the advantage of evaluating throughout the entire feeding event, it may not accurately reflect the infant’s intermittent stress signs during feeding [17].

There is a high prevalence of prematurity in Egypt, from 2.4% in 2011 to 4.7% in 2015. 61.3% of infants developed a poor fetal outcome, while 38.7% of infants had good fetal outcomes, contributing to neonatal feeding difficulties [21]. Unfortunately, feeding practices in the NICU are highly variable. There are no definitive criteria or specific policies for initiating oral feeding; rather, it primarily depends on the infant’s gestational age and weight. This encouraged us in previous research works to develop a new scoring system for early feeding skills in preterm infants (MEFSA) [22]. Also, an algorithm for the transition from tube feeding to oral feeding in preterm infants was developed [23].

The MEFSA [22] is an 85-item observational measure of oral feeding skill. It follows a cue-based feeding regimen. The MEFSA describes the feeding context (e.g., PMA on the day of assessment, current weight, baseline physiologic measures, need for supplemental oxygen, feeding route, feeding schedule, and prescribed volume). These descriptors are placed at the top of the assessment sheet. The MEFSA comprises three main sections: pre-feeding, during-feeding, and post-feeding sections. The “pre-feeding” section of MEFSA assesses four subsystems: (1) behavioral organization, (2) vital signs (Cardiopulmonary Stability) from a vital sign monitor and an oximeter, (3) reflexive oral motor skills, (4) non-nutritive sucking reflex. Followed by an assessment of nutritive sucking by NOMAS, then proceed to the next step during the feeding section. The “during feeding” section covers all stress signals that may appear during the feeding session. The items of the latter section are observational items that assess infant abilities to (a) remain engaged in feeding, (b) organize oral-motor skills, (c) maintain physiological stability, and (d) coordinate SSB. The “post-feeding” section takes 5 min to observe the infant and evaluate the impact of the feeding on the infant’s alertness, energy level, and physiological status. Moreover, recommendations are given to support the pre-existing feeding skills until systems are sufficiently mature for oral feeding. Also, interventions to facilitate the acquisition of OFS can be recommended. Finally, the MEFSA ends with a plan section for further follow-up assessment.

Therefore, the sections are complementary and cover the entire feeding task, as our role as a phoniatrician/speech language pathologist is to assess the infant before feeding, observe them during and after feeding, and help them bypass this critical period to achieve safe, efficient, and functional full oral feeding (Appendix-1).

An algorithm for transitioning from tube feeding to oral feeding in preterm infants [23] supports the hypothesis of early assessment of oral feeding skills (OFS) and early individualized support and feeding therapy if needed. It is a cue-based feeding approach. To practice functional oral feeding: (a) Initiate oral feeding once the infant is clinically stable, off the ventilator and/or CPAP, and has a functioning gastrointestinal system, (b) Follow a cue-based or infant-based technique that allows the infant to communicate, and according to the infant’s communication cues, the strategy is modified. (c) Use feeding competencies and assess the infant from moment to moment during feeding. (d) Most of those fragile preterm infants required individualized therapy.

Firstly, this study aimed to evaluate the accuracy of the pre-feeding section of the MEFSA [22] to initiate oral feeding in preterm infants and to identify a cutoff point for oral feeding readiness. Furthermore, to verify the agreement of the proposed cutoff point with the oral feeding skill level (OFS) scale and the success of the first oral feeding [20] as a gold standard objective tool. The second aim is to confirm the concordance between this score, which supports coordination safety, and the oral feeding skill level (OFS) [20] which supports the effectiveness.

## Subjects and methods

### Subjects

A transversal and analytical study was conducted in the NICU at Mansoura University Children’s Hospital (MUCH) and in the Phoniatrics unit at Mansoura University Hospitals. The Institutional Review Board (IRB) of the Faculty of Medicine approved the study protocol.

The research procedures were conducted under the principles of the Declaration of Helsinki [24] under the Proposal code MS.19.04.572. Informed consent was obtained from the legal guardians of all enrolled participants, ensuring confidentiality. Parents have the right to withdraw from the study at any time without penalty.

**Fig. 1:**
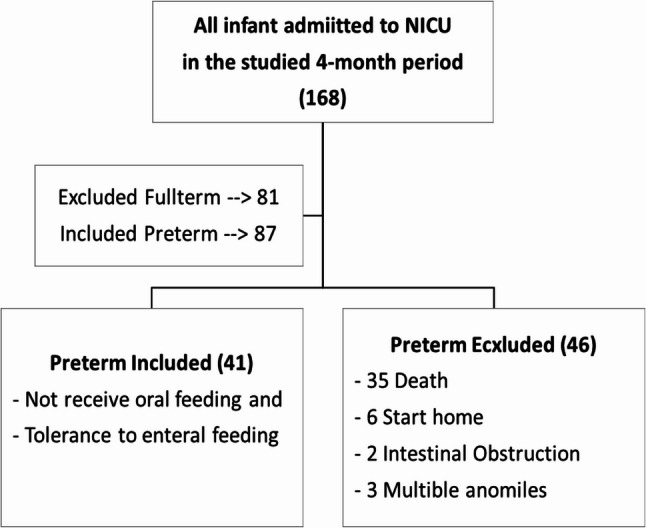
Study flow chart

All infants admitted to the NICU at MUCH during the studied period (four-month periods were chosen due to the study’s time constraints) were included or excluded according to the inclusion and exclusion criteria. Preterm infants who do not receive oral feeding and show tolerance to enteral feeding (pass meconium with audible intestinal sounds) were included in the sample. Infants who presented at least one of the following conditions were excluded from the study: a known congenital or chromosomal disease, cardiac malformation, infants who developed bronchopulmonary dysplasia, gastrointestinal problems (intestinal obstruction, feeding intolerance), head and neck malformation, or infants with intracranial hemorrhage or a surgical condition at the time of the study (Fig. 1).

## Methods

The study included 41 preterm infants. The assessments were conducted by a single, trained, and certified assessor (the first author), who had received standardized training in the use of the scale before the study. Her performance was supervised during pilot testing to ensure consistency. This procedure was intended to minimize assessment bias. Once the infant is diagnosed clinically stable by the attending neonatologist, off the ventilator and/or CPAP, and has a proven functioning GIT system, the assessment of the infant’s oral feeding readiness using the Pre-Feeding section of the MEFSA scale [22] was performed 15 min before the estimated time of the prescribed feeding session. This evaluation was conducted with the infants in a semi-reclined position, with their upper and lower limbs flexed and their head aligned with their spine. Using a proposed scale for assessment of oral feeding readiness and skills that consists of 5 domains: Scale of a behavioral organization, Scale of vital signs (Cardiopulmonary Stability), Scale of reflexive oral motor skills, Nonnutritive sucking (NNS) reflex for 1 min and then followed by Nutritive Sucking (NS) Reflex (2 min) by NOMAS [6, 7]. A score is given to each item of the protocol. The infant’s performance was determined by the sum of the scores obtained, which ranged from 20 to 87 (Appendix 1).

Subsequently, the level of oral feeding skills (OFS) [20] was evaluated during the first oral feeding. The procedure was carried out with the infants also in a semi-reclined position. The following parameters were observed: total volume of milk prescribed, volume of milk accepted during feeding, volume of milk accepted during the first 5 min of feeding, and duration of oral feeding. This information enabled the calculation of the proficiency and the milk transfer rate. The rate of milk transfer (RT) is monitored during the entire feeding process. The level of oral feeding skills was rated from 1 to 4 (Appendix 2) [20].

Moreover, during assessment by OFS, all infants were scored using the during-feeding section (Oral Feeding Maintenance) of the MEFSA [22] and followed the MEFSA oral feeding transition algorithm [23]. The During-feeding section of the MEFSA comprises 30 items proposed to observe the infant from moment to moment during feeding to assess the infant’s abilities to remain engaged in feeding, organize oral-motor skills, maintain physiological stability, and coordinate swallowing and breathing. A score is given to each item of the protocol. The performance of the infant was determined by the sum of the scores obtained, which ranged from 0 to 29, with an additional event, if present, subtracting one from the total score (Appendix 1).

The feeding assessment started at the introduction of the nipple of the bottle into the infant’s mouth and lasted for a maximum of 20 min. The process may be interrupted at the examiner’s discretion if signs of fatigue or stress are observed.

To address the aim of this study, we determined the overall diagnostic accuracy, sensitivity, and specificity of the MEFSA using the Receiver Operating Characteristics (ROC) curve approach. The success and oral feeding level obtained by the OFS score [20] in the first oral feeding, it was considered a gold standard to estimate the cutoff point, overall diagnostic accuracy, specificity, and sensitivity of the pre-feeding section of the MEFSA [22].

To address our second goal, i.e., to verify whether there is a concordance between the two assessment instruments, the results were dichotomized into “able to feed” and “unable to feed.” To be considered able to feed by MEFSA [22], infants should reach the previously estimated cutoff point of the pre-feeding section of the MEFSA. At the same time, assessing the level of oral feeding skills should be classified as level 4 or 3, i.e., proficiency higher than 30% is associated with an adequate ability to feed orally. Feeding is defined as successful if infants complete 80% of their prescribed feeding [20].

Furthermore, a Pearson correlation analysis was conducted between the total scores of the pre-feeding section and the during-feeding section of MEFSA. When the infant appears ready in the pre-feeding section, they safely pass to the during-feeding section.

Lastly, the association between the during-feeding section of MEFSA [22] and OFS [20] was examined using one-way analysis of variance (ANOVA) to determine whether there were any statistically significant associations between the means of two or more independent (unrelated) groups.

Data was entered into the computer and analyzed using the commercially available Statistical Package for the Social Sciences (IBM SPSS software package, version 24.0 for Windows).

## Results

The study included 41 preterm infants, with an average birth weight of 1747.0 ± 519.1 g and a gestational age of 32.8 ± 1.97 weeks. Regarding intrauterine growth, 82.9% of the sample was classified as appropriate for gestational age, 9.8% as small for gestational age, and 7.3% as large for gestational age (Table 1).

**Table 1:** Demographic data and baseline characteristics of the studied group (*N* = 41)

Parameters	The studied group (*N* = 41)
Gestational age *(days)*	229.8 ± 13.8
*(weeks)*	32.8 ± 1.97
Pre-maturity class	
➢Mild preterm (36 w-	27 (65.9%)
➢Very preterm (32 w-	11 (26.8%)
➢Extremely preterm (≤ 28 w)	3 (7.3%)
Birth/admission weight *(g)*	1747.0 ± 519.1
Body weight categories	
➢Normal	4 (9.8%)
➢LBW	21 (51.2%)
➢VLBW	13 (31.7%)
➢ELBW	3 (7.3%)
Weight percentile age	
➢Appropriate for the age	34 (82.9%)
➢Not appropriate for the age	7 (17.1%)
• Small for age	4 (9.8%)
• Large for age	3 (7.3%)
Needing Ventilator and/or CPAP	
➢No	13 (31.7%)
➢Yes	28 (68.3%)
Off Ventilator and/or CPAP and showing tolerance to enteral feeding PMA *(days)*	233.9 ± 15.3
*(weeks)*	33.1 ± 2.18

Data expressed as mean ± SD or number

The clinical assessments were performed when the infants had a medical prescription for enteral feeding. At the time of evaluation, the preterm infants had an average postmenstrual age (PMA) of 33.74 ± 1.87 weeks and a weight of 1683.7 ± 429.9 g. The MEFSA scoring results: mean total score of the pre-feeding Scale was 80.2 ± 4.5 (67–87) out of (20:87), 63.4% of infants had disorganized sucking pattern by NOMAS, and the mean total score of during feeding scale was 27.2 ± 2.2 (21–29) out of (0:29) (Table 2).

**Table 2:** MEFSA scoring (*N* = 41)

Pre-Feeding Scale (Oral Feeding Readiness & OFS) of the intervention group	
Parameters	*N* = 41
Scale of behavioral organization *(7:21)*	19.8 ± 1.6 (15–21)
Scale of vital signs (Cardiopulmonary Stability) *(5:15)*	14.7 ± 0.84 (11–15)
Scale reflexive oral motor skills *(8:24)*	22.3 ± 1.7 (16–24)
Scale of non-nutritive sucking reflex *(0:27)*	23.3 ± 2.8 (19–27)
Total score of Pre-Feeding Scale *(20:87)*	80.2 ± 4.5 (67–87)
Nutritive Sucking (NOMAS)	
➢ Normal	13 (31.7%)
➢ Disorganized	26 (63.5%)
➢ Dysfunctional	1 (2.4%)
➢ Absent	1 (2.4%)

During feeding (Oral Feeding Maintenance) Scale	
Parameters	*N* = 40 #
Maintain Engaged in Feeding *(0:4)*	4 (2–4)
Maintain Vital Signs (Cardiopulmonary Stability) *(0:4)*	4 (1–4)
Other Clinical Difficulties *(0:22)*	
➢ Respiratory difficulties *(0:4)*	4 (2–4)
➢ Swallowing difficulties *(0:5)*	5 (1–5)
➢ Visceral response *(0:4)*	4 (2–4)
➢ Motor response *(0:5)*	5 (3–5)
➢ Facial or ocular response *(0:3)*	3 (2–3)
Total score of the During Feeding Scale *(0:29)*	27.2 ± 2.2 (21–29)

Data expressed as mean ± SD, median (minimum–maximum), or number (%)

#: The baby with absent Nutritive Sucking was excluded

**Fig. 2:**
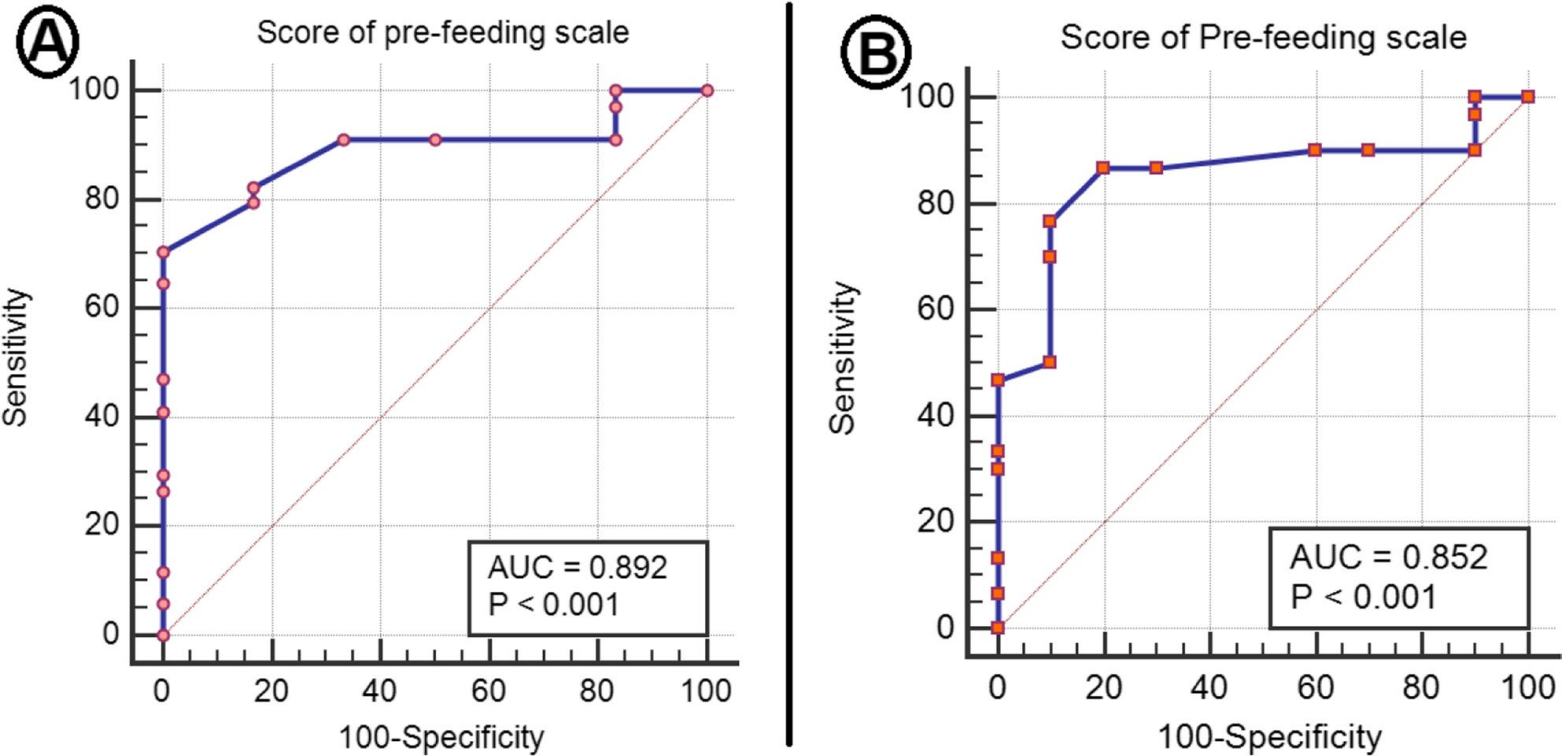
ROC in relation to (**A**) oral feeding skill level and (**B**) oral feeding success

To determine the cutoff point of the oral feeding readiness section of MEFSA concerning OFS, a ROC curve for the total score of the pre-feeding scale concerning oral feeding skill level was summarized in (Table 3) and (Fig. 2).

**Table 3:** ROC curve for the pre-feeding scale of MEFSA

	AUC	95% CI		Cutoff	Sensitivity	Specificity	PPV	NPV	Accuracy
		Lower	Upper						
Score of the pre-feeding scale	ROC curve in relation to oral feeding skill level								
	0.89	0.79	0.99	> 77	82.4%	83.3%	96.6	45.5	82.5%
	ROC curve in relation to oral feeding success								
	0.85	0.73	0.98	> 77.5	86.7%	70%	89.7	63.6	82.5%

*AUC* area under the curve, *CI* confidence interval, *PPV* positive predictive value, *NPV* negative predictive value

Concerning the oral feeding skill level of OFS, a cutoff point of oral feeding readiness > 77 out of a total of 87 points was determined. Regarding the oral feeding success of OFS, a cutoff point of > 77.5 out of 87 points was determined. The overall diagnostic accuracy obtained was 82.5%. The sensitivity and specificity concerning the oral feeding skill level of OFS were 82.4% and 83.3%, respectively. Moreover, the sensitivity and specificity concerning the oral feeding success of OFS were 86.7% and 70%, respectively.

The area under the ROC curve for oral feeding skill level was 0.89, while for oral feeding success, it was 0.85, indicating that MEFSA has moderate to high accuracy in evaluating readiness for SOF.

The concordance between the two assessment instruments was evaluated in terms of the identification of infants as able to feed (levels 4 and 3) or unable to feed (levels 2 and 1). It was verified that 82.35% of the preterm infants considered able to feed by the quantitative protocol OFS were also regarded as able to feed by the pre-feeding section of the MEFSA (scored more than the proposed cutoff point > 77). Additionally, 83.33% of the infants considered unable to feed by the quantitative protocol OFS were also regarded as unable by the pre-feeding section of the MEFSA (scored equal or less than the proposed cutoff point < 77) (Table 4 - section A). In general, both protocols agreed on the assessment of 82. 5% (*N* = 33) of the sample of preterm infants (*N* = 40). One infant with an absent NS, as per NOMAS, is not included in the assessment.

**Table 4:** Distribution of preterm infants regarding the results obtained with both assessment protocols, considering the dichotomization of able and unable to feed

A. In relation to oral feeding skill level.			
Pre-feeding of MEFSA	Level of oral feeding skill		Total *n* (%)
	Able *n* (%)	Unable *n* (%)	
Able	28 (82.35%)	1 (16.67%)	29 (100%)
Unable	6 (17.65%)	5 (83.33%)	11 (100%)
Total	34 (100%)	6 (100%)	40 (100%)

B. In relation to oral feeding success.			
Pre-feeding of MEFSA	Oral feeding success		Total *n* (%)
	Able *n* (%)	Unable *n* (%)	
Able	26 (86.67%)	3 (30%)	29 (100%)
Unable	4 (13.33%)	7 (70%)	11 (100%)
Total	34 (100%)	6 (100%)	40 (100%)

The studied infants were categorized as either able to feed (successful oral feeding; infants took >80% of the prescribed volume) or unable to feed (unsuccessful oral feeding; infants took <80% of the prescribed volume). It was verified that 86.67% of the preterm infants considered able to feed by the quantitative protocol OFS were also regarded as able to feed by the pre-feeding section of the MEFSA (scored > 77). Additionally, 70% of the infants considered unable to feed by the quantitative protocol OFS were also regarded as unable by the pre-feeding section of the MEFSA (scored < 77) (Table 4, Section B). In general, both protocols agreed on the assessment of 82. 5% (*N* = 33) of the sample of preterm infants (*N* = 40). One infant with an absent NS by NOMAS is excluded from the 1st oral feeding assessment.

Moreover, the Pearson correlation between the total scores of the pre-feeding section and the during-feeding section of MEFSA was obtained. Table 5 showed a moderate positive correlation coefficient between the pre-feeding and during-feeding sections, with a statistically significant correlation.

**Table 5:** Pearson correlation between MEFSA pre-feeding and during-feeding sections

Scores	*R*	*P*
Total score of Pre-Feeding Scale - Total score of During Feeding Scale	0.47	0.002*

*: significant *p* ≤ 0.05. r: correlation coefficient

Finally, a statistically significant association was found between the during-feeding section of MEFSA (infant-based assessment) and both oral feeding success and oral feeding skill level, as measured by OFS (volume-based assessment) (Table 6).

**Table 6:** Association between score of during the feeding section of MEFSA and OFS score (*n* = 40)

OFS Items	During-feeding score	Test of significance
Oral feeding success		
➢ No (*n* = 10)	25.2 ± 2.9	**t = 3.9**
➢ Yes (*n* = 30)	27.9 ± 1.4	***p*** = 0.001*
OFS level		
➢ OFS level 1 (*n* = 4)	23 ± 1.4	**F = 12.2** ***P*** ≤ 0.001*
➢ OFS level 2 (*n* = 2)	25 ± 5.7	
➢ OFS level 3 (*n* = 4)	27.5 ± 1	
➢ OFS level 4 (*n* = 30)	27.9 ± 1.3	

Data expressed as mean ± SD

t: independent samples -t-test

F: one-way ANOVA test

*: significant *p* ≤ 0.05

## Discussion

In previous research works, a novel scale, “the MEFSA Scale” [22], and an algorithm for the transition from tube feeding to oral feeding were developed [23]. In this study, we assessed the accuracy of the designed scoring scale [22]. Specifically, the pre-feeding section on the initiation of oral feeding in preterm infants identified a cutoff point for readiness to initiate oral feeding.

The pre-feeding section supports the growing body of research on oral feeding readiness, which defines readiness as a complex concept that can serve as an indicator of an infant’s feeding emergence, ability to feed orally, and readiness for a particular feeding event. In our analysis, the pre-feeding section of MEFSA is a straightforward and quick instrument that can be utilized in clinical practice. The results of the MEFSA study, using the Receiver Operating Characteristics (ROC) analysis, indicate that MEFSA had a moderate-to-high accuracy in evaluating readiness to SOF with a cutoff point of oral feeding readiness greater than 77.

Our study showed better results in comparison to the de Paula Bolzan et al. study [19] who assessed preterm infants by the POFRAS regarding their readiness to initiate oral feeding and by the OFS level [20] during the first oral feeding. De Paula Bolzan et al. study [19] showed an overall diagnostic accuracy of 71.3%, and a sensitivity and a specificity of 61.36% and 78.95%, respectively, while the area under the ROC curve was 0.71. Furthermore, agreement of the proposed cutoff point with the OFS level scale [20] and the success of the first oral feeding was verified. Regarding both OFS level and oral feeding success, both protocols agreed on the assessment of 82. 5%, and that is better than the results of the de Paula Bolzan et al. study [19] that showed 65.85% agreement between the two protocols.

The readiness to feed orally, based on behavioral measures, may not guarantee success in oral feeding [19]. As in nutritive sucking, other aspects are relevant, especially the coordination between sucking, swallowing, and breathing [20]. It was the limitation of the de Paula Bolzan et al. study [19] that faced in their POFRAS score. Hence, we added an assessment of nutritive sucking by NOMAS [10, 11], then proceeded to the next step in the during-feeding section.

The during-feeding section supports infant-based feeding, allowing the infant to communicate and express stress signals that must be respected. As reported by de Paula Bolzan et al. [19], fatigue signs are likely minimal at the first five minutes of oral feeding assessment. Therefore, the identification of infants with low fatigue with oral feeding using only the pre-feeding section and nutritive sucking by NOMAS for 2 min, is somewhat complex and insufficient. The literature suggests that an infant who becomes fatigued during feeding may score lower on remaining engaged and may exhibit less coordinated swallowing and breathing due to decreased arousal, despite having a low fatigue score and a high score on pre-feeding assessment [25].

The pre-feeding and during-feeding sections outlined essential aspects of pre-term infants’ feeding behavior, which, when analyzed together, facilitate a quicker and more effective transition to oral feeding. Our study revealed a positive and statistically significant correlation between the pre-feeding and during-feeding sections, indicating that when an infant is ready by the pre-feeding section, they safely progress through the during-feeding section.

There are two dilemmas faced when assessing oral feeding maintenance: coordination, safety, and feeding effectiveness. The second aim of this study was to confirm the concordance between the MEFSA [22] which supports coordination, safety, and the OFS level [20] which supports the effectiveness. This study showed a statistically significant association between the MEFSA and the OFS. Therefore, this section supports the safety infant-based approach, while also achieving the effectiveness of the volume-based approach.

Diagnostic accuracy is an essential element in healthcare decision-making. Therefore, an instrument with 82.5% accuracy, such as the pre-feeding section of MEFSA, should be used with caution. A combination of pre-feeding and during-feeding sections is preferred to improve the introduction of oral feeding in preterm infants and address this issue. The pre-feeding section assesses readiness to feed orally, based only on behavioral measures and reflexive oral sensory-motor skills. However, it may not guarantee successful and safe oral feeding. The during-feeding section of the MEFSA can assess the oral feeding maintenance, which is relevant to describe successful, effective, and safe oral feeding.

It is worth mentioning that using the NOMAS protocol is not mandatory in the assessment by the MEFSA. NOMAS is not a main section in MEFSA and is not a part of the scoring system. The MEFSA is sufficient on its own, and the NOMAS can be used as a complementary step. The pre-feeding section of MEFSA assesses the infant’s readiness to start oral feeding. According to the proposed cut-off for the Pre-Feeding section, if the infant passes the cutoff (> 77), the infant is labeled as being ready to SOF, and the clinician could proceed to the next step. If the clinician is certified with NOMAS, he/she can use it to assess the NS and recommend specific strategies such as pacing, regulation, positioning, changing flow rate, and/or oral support. Then the clinician would start the during-feeding section while using the recommended strategies. If the clinician who conducts the MEFSA is not NOMAS certified, he can proceed directly to the during feeding section. The during-feeding section covers all important items that assess the coordination and maintenance of NS, and if any stress cues are observed, specific strategies would be used immediately.

The integration of the MEFSA as a bedside assessment tool for early oral feeding skills in NICUs will lead to infants who are successful feeders, not just those who are successfully fed. Once any infant becomes clinically stable, off the ventilator and/or CPAP, and has a proven functioning GIT system, the clinician can use the MEFSA to assess the infant’s first oral feeding according to the proposed cut-off of the Pre-Feeding section (> 77). Again, if the infant passes the cutoff, they are labeled as ready to SOF, and the clinician can proceed to the next during-feeding and post-feeding sections. If not passed the cutoff point, the infant is described as not ready to SOF and Ryle feeding with oral stimulation strategies would be recommended till the follow-up after 72 h. Regarding the during-feeding section, if any stress signal is observed, immediate intervention will be used to evaluate whether it is sufficient to provide safe oral feeding. Accordingly, recommendations will be made for safe SOF with specific strategies or for delaying SOF with oral motor stimulation until the next follow-up after 72 h.

**Fig. 3:**
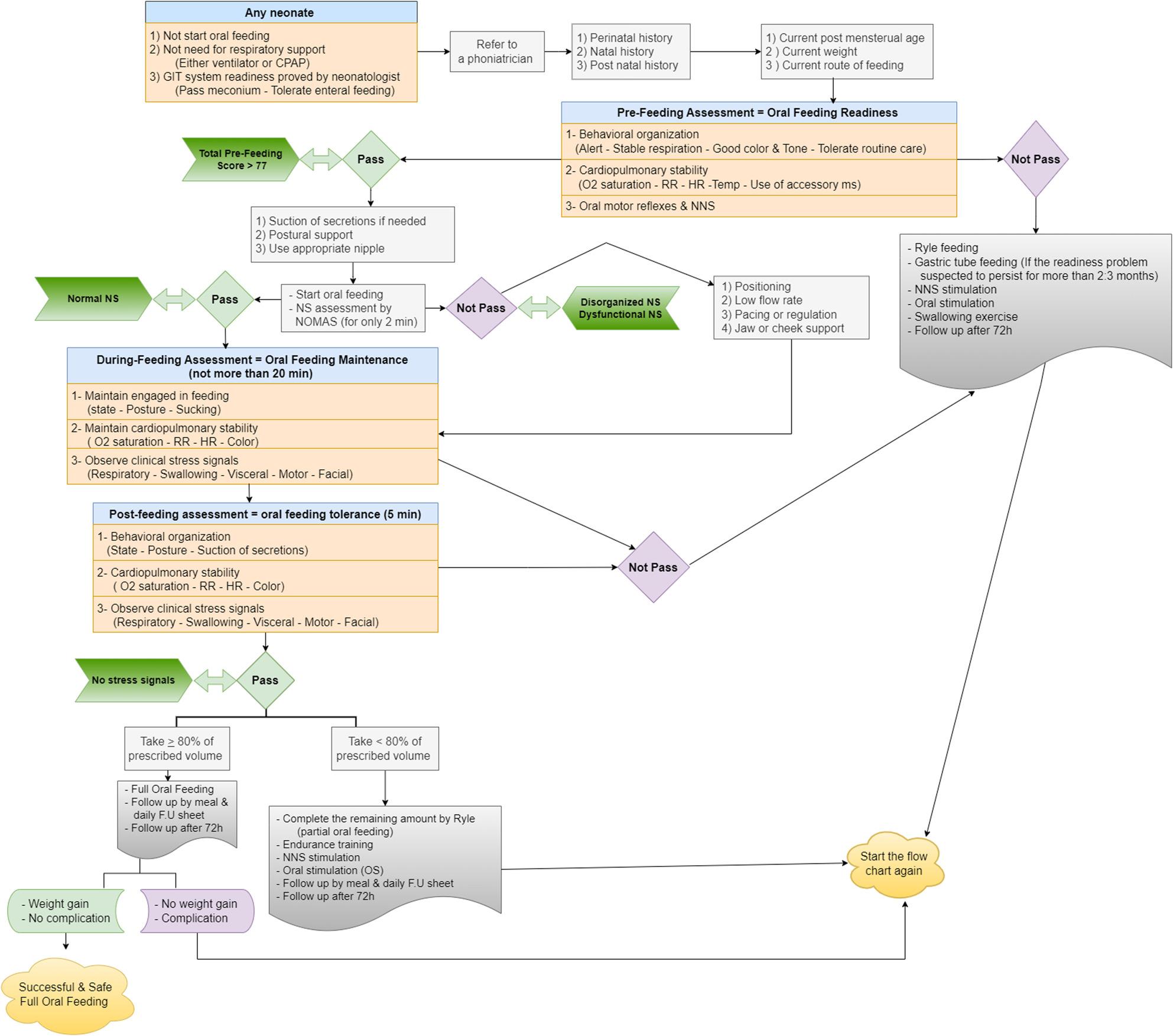
An algorithm for assessing and supporting oral feeding in preterm infants following the MEFSA score

Therefore, according to the MEFSA, to describe the infant as being ready or not, they should score more than the proposed cutoff point of the pre-feeding section (> 77). If the infant passes the cutoff, proceed to the next during-feeding section. If not passed the cutoff point, oral stimulation strategies would be recommended till the subsequent follow-up. The proposed novel algorithm for oral feeding (Fig. 3) is the most significant contribution of this study in assessing and supporting oral feeding in preterm infants.

### Limitations

This study faced some limitations, such as the small sample size and potential observer bias. Furthermore, this study was conducted at a single center; therefore, the findings cannot be generalized to the general population.

### Conclusion and future directions

The pre-feeding section of MEFSA is a straightforward and quick instrument that can be utilized in clinical practice. It was better to increase the sample size and generalize the use of the MEFSA in more NICUs. It would enhance and fortify the evidence for this research. For future research, the application of the MEFSA in preterm infants while still on non-invasive respiratory support, such as nasal continuous positive airway pressure (CPAP), should be explored. Another suggested area for future research is to examine which MEFSA items can distinguish between preterm infants who demonstrate readiness for oral feeding and those with insufficient readiness cues.

## Supplementary Information

Supplementary Material 1: Appendix 1: Mansoura Early Feeding Skills Assessment (MEFSA) Score. Appendix 2: A standardized Oral Feeding Skill (OFS) level evaluation tool.

## Data Availability

available (The datasets used and/or analyzed during the current study are available from the corresponding author).

## Abbreviations

AUC: Area under the curve
CI: Confidence interval
CPAP: Continuous Positive Airway Pressure
EFS: Early Feeding Skills Assessment
ELBW: Extreme Low Birth-Weight
IRB: Institutional Research Board
LBW: Low Birth-Weight
MEFSA: Mansoura Early Feeding Skills Assessment
MUCH: Mansoura University Children's Hospital
NPV: Negative predictive value
NICU: Neonatal Intensive Care Unit
NOMAS: Neonatal Oral Motor Assessment Scale
NNS: Nonnutritive sucking
NS: Nutritive Sucking
ANOVA: One-way analysis of variance
OFS: Oral feeding skill
PPV: Positive predictive value
PMA: Post Menstrual Age
POFRAS: Premature Oral Feeding Readiness Assessment Scale
PINFRS: Preterm Infant Nipple Feeding Readiness Scale
RT: Rate of milk Transfer
ROC: Receiver Operating Characteristics
SD: Standard Deviation
VLBW: Very Low Birth-Weight
