# Supplementary material for: Accuracy of Mansoura Early Feeding Skills Assessment score (MEFSA) in identification of oral feeding readiness in preterm infants
Source: BMC Pediatr. 2026 Mar 18;26:281. doi: 10.1186/s12887-026-06586-z (PMC13063901; doi:10.1186/s12887-026-06586-z)

**Appendix 1**

**Mansoura Early Feeding Skills Assessment (MEFSA) Score**

**مقياس المنصورة لتقييم مهارات التغذية للرضع**

Pre-Feeding Scale

(Oral Feeding Readiness & Oral Feeding Skills)

| **Scale of behavioral organization** | **3** | | | **2** | | | **1** | | | |
| --- | --- | --- | --- | --- | --- | --- | --- | --- | --- | --- |
| - **Behavioral State** | Alert | | | Sleep | | | Crying or Drowsy | | | |
| - **Respiration** | Room air | | | O2 in incubator | | | Nasal O2 cannula | | | |
| - **Skin Color** | Normal | | | Jaundice | | | - Cyanosis | - Pallor | | |
| - **Need of Secretion Aspiration** | Unnecessary | | | Seldom (1:2/day) | | | Frequent (>2/day) | | | |
| - **Routine Care Handling** | Tolerate | | | Just Irritability | | | Couldn't Tolerate | | | |
| - **Global Posture** | Flexed | | | Partially flexed | | | Extended | | | |
| - **Global Tonus** | Normotonic | | |  | | | - Hypotonia | | | - Hypertonia |
|  | | | | | | | | | | |
| **Scale of vital signs (Cardiopulmonary Stability)** | | **3** | | | | **1** | | | | |
| - **O2 saturation** | | > 92 % **(Basal =** …….**)** | | | | < 92 % | | | | |
| - **Respiratory Rate** | | < 60 beat/min **(Basal =** …......**)** | | | | > 60 beat/min | | | | |
| - **Use of accessory muscle** | | No use | | | | Suprasternal or Subcostal retraction | | | | |
| - **Heart Rate** | | 100 :180 b/min **(Basal =** …..**)** | | | | - Tachycardia | | - Tachypnea | | |
| - **Temperature** | | Normal | | | | - Fever | | - Hypothermia | | |
|  | | | | | | | | | | |
| **Scale of** **Reflexive Oral Motor Skills** | | | **3** | | **1** | | | | | |
| - **Transverse tongue reflex** | | | Goal directed | | Non-goal directed | | | | No response | |
| - **Tongue reflex** | | | Normal | | Absent | | | | | |
| - **Biting reflex** | | | Normal | | Exacerbated (Tonic bite) | | | | | |
| - **Grasp reflex** | | | Present | | Absent | | | | | |
| - **Palmo-mental reflex** | | | Present | | Absent | | | | | |
| - **Perioral sensitivity** | | | Present | | Exaggerated | | | | Absent | |
| - **Intra-oral sensitivity (Gag reflex)** | | | Present | | Exaggerated | | | | Absent | |
| - **Rooting reflex** | | | Effective | | Absent or need encouragement | | | | | |

|  | | | | | | | |
| --- | --- | --- | --- | --- | --- | --- | --- |
| **Non-Nutritive Sucking (1min)** | **3** | | **1** | | | **0** | |
| - **Easy initiation of sucking** | Spontaneous | | Need stimulation | | | Absent | |
| - **Ability to latch (labial sealing)** | Good | | Poor | | |  |  |
| - **Coordination () lip, tongue & jaw** | Good | | Poor | | |  |  |
| - **Suck – pause** | 5:8 | | >8 <5 | | |  |  |
| - **Rate** | 2/sec | | More or less | | |  |  |
| - **Strain** | Strain | | Weak | | |  |  |
| - **Stress signals** | Absent | | Present | | |  |  |
| - **Habituation** | Absent | | Present | | |  |  |
| - **Preservation** | Absent | | Present | | |  |  |
|  | | | | | | | |
| **Nutritive Sucking Reflex (2min) By NOMAS** | | Normal | | Disorganized | Dysfunctional | | Absent |

During Feeding

(Oral Feeding Mai**ntenanc**e)

| **Maintain Engaged in Feeding** | | **1** | **0** |
| --- | --- | --- | --- |
| - **State** | | Maintain | Not maintain |
| - **Global posture** | | Maintain | Not maintain |
| - **Sucking (Force & Rhythm)** | | Maintain | Not maintain |
| - **Refuse of food & Gaze aversion (looking away)** | | No | Yes |
|  | |  | |
| **Maintain Vital Signs (Cardiopulmonary Stability)** | | **1** | **0** |
| - **O2 saturation drops < 90 %** | | No | Yes |
| - **Respiratory Rate (Rise or Drop)** | | No | Yes |
| - **Heart Rate (Rise 15 beats above baseline or Drops < 100 b/min)** | | No | Yes |
| - **Skin color changes (Cyanosis or Pallor or Flushing)** | | No | Yes |
|  | |  |  |
| **Other Clinical Symptoms** | | **1** | **0** |
| **Respiratory difficulties** | - **Apnea or stop sucking to breath** | No | Yes |
|  | - **Stridor or Grunting (Any noisy breathing)** | No | Yes |
|  | - **Dyspnea & Substernal or suprasternal retraction** | No | Yes |
|  | - **Nasal flaring** | No | Yes |
| **Swallowing difficulties** | - **Coughing / Chocking** | No | Yes |
|  | - **Multiple swallows to clear a bolus** | No | Yes |
|  | - **Wet voice or gurgling sound** | No | Yes |
|  | - **Nasal spit up** | No | Yes |
|  | - **Bolus stagnation & Milk Drooling** | No | Yes |
| **Visceral response** | - **Gaging & Vomiting** | No | Yes |
|  | - **Hiccups** | No | Yes |
|  | - **Sneezing** | No | Yes |
|  | - **Yawning** | No | Yes |
| **Motor response** | - **Arching** | No | Yes |
|  | - **Frantic flailing movement (position & baby support)** | No | Yes |
|  | - **Finger or toes splaying** | No | Yes |
|  | - **Salute (hand blocking face)** | No | Yes |
|  | - **Epileptic attack or Tremors** | No | Yes |
| **Facial or Ocular response** | - **Glassy eye (tuning out) & Staring** | No | Yes |
|  | - **Panicked or painful look** | No | Yes |
|  | - **Grimacing or frowning** | No | Yes |
| **Others** | - **Any other findings ……………………………….** | For one present 🡪 -1 | |

Oral feeding skill level

| - **Overall transfer** | $(volume taken)/(total perscribed )$ | | **………. /………… =** |
| --- | --- | --- | --- |
| - **PRO** | $(volume in 1st 5min)/(total perscribed )$ | | **………. /………… =** |
| - **RT** | $(volume taken)/(time )$ | | **………. /………… =** |
| - **Success** | |  | |
| - **Oral feeding skill level** | |  | |

1^st^ **5min post-feeding**

(Oral Feeding Tolerance)

| **Behavioral Organization** | | **1** | | **0** | | |
| --- | --- | --- | --- | --- | --- | --- |
| - **State** | | Sleepy or Calm | | Drowsy | Crying & Irritable | |
| - **Global posture** | | Same as pre-feeding | | Changed | | |
| - **Need of secretion aspiration** | | Same as pre-feeding | | Increase need | | |
|  | | | | | | |
| **Vital Signs (Cardiopulmonary Stability)** | | **1** | | **0** | | |
| - **O2 saturation** | | Same as pre-feeding | | Changed | | |
| - **Respiratory Rate** | | Same as pre-feeding | | Changed | | |
| - **Heart Rate** | | Same as pre-feeding | | Changed | | |
| - **Skin color changes (Cyanosis or Pallor or Flushing)** | | No | | Yes | | |
|  | | | | | | |
| **Other Clinical Difficulties** | | | **1** | | | **0** |
| **Respiratory difficulties** | - **Apnea** | | No | | | Yes |
|  | - **Stridor or Grunting (Any noisy breathing)** | | No | | | Yes |
|  | - **Dyspnea & Substernal or suprasternal retraction** | | No | | | Yes |
|  | - **Nasal flaring** | | No | | | Yes |
| **Swallowing difficulties** | - **Coughing / Chocking** | | No | | | Yes |
|  | - **Nasal spit up** | | No | | | Yes |
|  | - **Wet voice or gurgling sound** | | No | | | Yes |
| **Visceral response** | - **Vomiting** | | No | | | Yes |
|  | - **Hiccups** | | No | | | Yes |
|  | - **Abnormal colonic movement or passing gas** | | No | | | Yes |
|  | - **Sneezing** | | No | | | Yes |
| **Motor response** | - **Arching** | | No | | | Yes |
|  | - **Frantic flailing movement** | | No | | | Yes |
|  | - **Epileptic attack or Tremors** | | No | | | Yes |
|  | - **Salute (hand blocking face)** | | No | | | Yes |
| **Facial or Ocular response** | - **Glassy eye (tuning out) & Staring** | | No | | | Yes |
|  | - **Panicked or painful look** | | No | | | Yes |
| **Others** | - **Any other findings …………………………………………...** | | For one present 🡪 -1 | | | |

Recommendation

| - **Oral or Ryle feeding** |  |
| --- | --- |
| - **Nipple level** |  |
| - **Strategy during feeding** |  |
| - **Stimulation** |  |

**Appendix 2**

**A standardized Oral Feeding Skill (OFS) level evaluation tool**

- **Proficiency (PRO)** is %ml taken during the first 5 min/total ml prescribed. It is reflective of infants’ actual feeding skills are that when fatigue is minimal (< 30% or ≥ 30%)
- Proficiency (PRO) (volume in 1st 5min) / (total prescribed}.
- **Rate of milk transfer (RT)** reflects their overall skills when fatigue comes into play (ml/min)
- Rate of transfer (RT) (volume taken) / (time).
- **Overall transfer or Feeding performance** **(OT)** reflects feeding success and equals %ml taken during a feeding/total ml prescribed (No < 80% or yes ≥ 80%)
- Overall transfer (volume taken) / (total prescribed).
- **Oral feeding skill levels (OFS)**: Four OFS levels, as defined by infant actual feeding skills (PRO) and endurance (RT), include level 1, level 2, level 3, or level 4.


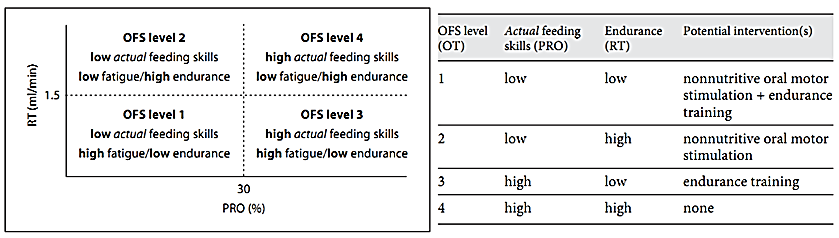

Supplement: Supplementary file 1 — Supplementary Material 1: Appendix 1: Mansoura Early Feeding Skills Assessment (MEFSA) Score. Appendix 2: A standardized Oral Feeding Skill (OFS) level evaluation tool. [file 12887_2026_6586_MOESM1_ESM.docx]
